# *catena*-Poly[[[bis­(3-amino­pyrazine-2-carboxyl­ato)triaqua­praseodymium(III)]-μ-3-amino­pyrazine-2-carboxyl­ato-[(3-amino­pyrazine-2-carboxyl­ato)diaqua­formatopraseodymium(III)]-μ-3-amino­pyrazine-2-carboxyl­ato] hexa­hydrate]

**DOI:** 10.1107/S1600536811031308

**Published:** 2011-08-11

**Authors:** Shan Gao, Seik Weng Ng

**Affiliations:** a Key Laboratory of Functional Inorganic Material Chemistry, Ministry of Education, Heilongjiang University, Harbin 150080, People’s Republic of China; b Department of Chemistry, University of Malaya, 50603 Kuala Lumpur, Malaysia; c Chemistry Department, Faculty of Science, King Abdulaziz University, PO Box 80203 Jeddah, Saudi Arabia

## Abstract

The asymmetric unit of the polymeric title compound, {[Pr_2_(C_5_H_4_N_3_O_2_)_5_(CHO_2_)(H_2_O)_5_]·6H_2_O}_*n*_, has two independent Pr^III^ atoms; one is coordinated by two water mol­ecules and the other by three water mol­ecules. The first is *N*,*O*-chelated by three 3-amino­pyrazine-2-carboxyl­ate ions, whereas the second is chelated by two carboxyl­ate ions; both exist in a monocapped square-anti­prismatic geometry. The polymeric chains that run along the *a* axis inter­act with the lattice water mol­ecules, generating a three-dimensional hydrogen-bonded network. The formate ion is disordered over two positions with respect to the non-coordinated atoms in a 1:1 ratio.

## Related literature

3-Amino­pyrazine­carb­oxy­lic acid decomposition with subsequent oxalate formation has been documented in a related lanthanum system; see: Gao & Ng (2011 ▶).
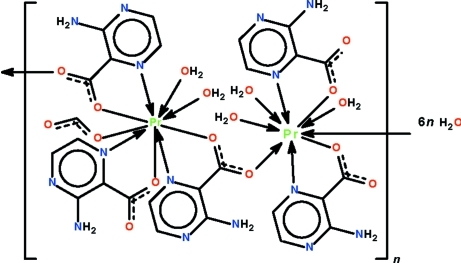


## Experimental

### 

#### Crystal data

[Pr_2_(C_5_H_4_N_3_O_2_)_5_(CHO_2_)(H_2_O)_5_]·6H_2_O
*M* *_r_* = 1215.58
Triclinic, 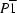

*a* = 9.7213 (3) Å
*b* = 14.2113 (6) Å
*c* = 17.6228 (6) Å
α = 68.801 (1)°
β = 76.291 (1)°
γ = 79.349 (1)°
*V* = 2191.97 (14) Å^3^
*Z* = 2
Mo *K*α radiation
μ = 2.30 mm^−1^
*T* = 293 K
0.14 × 0.12 × 0.07 mm

#### Data collection

Rigaku R-AXIS RAPID IP diffractometer
Absorption correction: multi-scan (*ABSCOR*; Higashi, 1995 ▶) *T*_min_ = 0.739, *T*_max_ = 0.856
21572 measured reflections
9906 independent reflections
8214 reflections with *I* > 2σ(*I*)
*R*_int_ = 0.041

#### Refinement

*R*[*F* ^2^ > 2σ(*F* ^2^)] = 0.035
*wR*(*F* ^2^) = 0.088
*S* = 1.06
9906 reflections
679 parameters
69 restraints
H atoms treated by a mixture of independent and constrained refinement
Δρ_max_ = 1.34 e Å^−3^
Δρ_min_ = −1.03 e Å^−3^

### 

Data collection: *RAPID-AUTO* (Rigaku, 1998 ▶); cell refinement: *RAPID-AUTO*; data reduction: *CrystalClear* (Rigaku/MSC, 2002 ▶); program(s) used to solve structure: *SHELXS97* (Sheldrick, 2008 ▶); program(s) used to refine structure: *SHELXL97* (Sheldrick, 2008 ▶); molecular graphics: *X-SEED* (Barbour, 2001 ▶); software used to prepare material for publication: *publCIF* (Westrip, 2010 ▶).

## Supplementary Material

Crystal structure: contains datablock(s) global, I. DOI: 10.1107/S1600536811031308/xu5282sup1.cif

Structure factors: contains datablock(s) I. DOI: 10.1107/S1600536811031308/xu5282Isup2.hkl

Additional supplementary materials:  crystallographic information; 3D view; checkCIF report

## Figures and Tables

**Table 1:** Hydrogen-bond geometry (Å, °)

*D*—H⋯*A*	*D*—H	H⋯*A*	*D*⋯*A*	*D*—H⋯*A*
O1w—H11⋯O7^i^	0.84 (1)	2.34 (2)	3.115 (4)	155 (5)
O1w—H12⋯O2^ii^	0.84 (1)	1.80 (1)	2.635 (5)	179 (5)
O2w—H21⋯O6w	0.84 (1)	2.02 (2)	2.839 (5)	164 (5)
O2w—H22⋯O6	0.84 (1)	1.99 (3)	2.771 (4)	153 (5)
O3w—H31⋯O7^i^	0.84 (1)	2.05 (2)	2.829 (4)	155 (4)
O3w—H32⋯O7w	0.84 (1)	1.89 (1)	2.721 (5)	174 (4)
O4w—H41⋯O8w^iii^	0.84 (1)	1.93 (1)	2.769 (5)	176 (6)
O4w—H42⋯O12^iv^	0.84 (1)	2.07 (5)	2.649 (7)	126 (5)
O5w—H51⋯O6w^iii^	0.84 (1)	1.97 (1)	2.803 (5)	174 (5)
O5w—H52⋯O11w^v^	0.84 (1)	1.84 (1)	2.673 (5)	173 (6)
O6w—H61⋯N11^vi^	0.84 (1)	2.02 (2)	2.842 (5)	169 (6)
O6w—H62⋯O10	0.84 (1)	2.02 (2)	2.833 (5)	164 (6)
O7w—H71⋯O9	0.84 (1)	2.41 (4)	3.135 (6)	146 (7)
O7w—H72⋯O12′	0.84 (1)	1.99 (5)	2.688 (10)	141 (8)
O8w—H81⋯O7w	0.84 (1)	2.01 (3)	2.782 (6)	154 (7)
O8w—H82⋯N2^vii^	0.84 (1)	2.03 (2)	2.861 (6)	168 (7)
O9w—H91⋯O10w^viii^	0.84 (1)	2.40 (6)	3.074 (8)	138 (7)
O10w—H102⋯N5^ix^	0.84 (1)	2.11 (3)	2.893 (6)	155 (7)

Symmetry codes: (i) 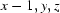; (ii) 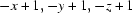; (iii) 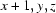; (iv) 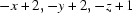; (v) 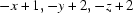; (vi) 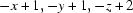; (vii) 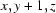; (viii) 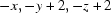; (ix) 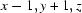.

**Fig. 1.**
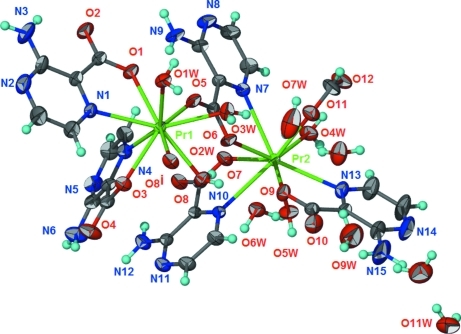
Thermal ellipsoid plot (Barbour, 2001) of the asymmetric unit of polymeric Pr2(H2O)5(CHO2)(C5H4N3O2)5.6H2O at the 50% probability level; hydrogen toms are drawn as spheres of arbitrary radius. Carbon atoms are not labeled. Symmetry code: i = x – 1, y, z.

**Fig. 2.**
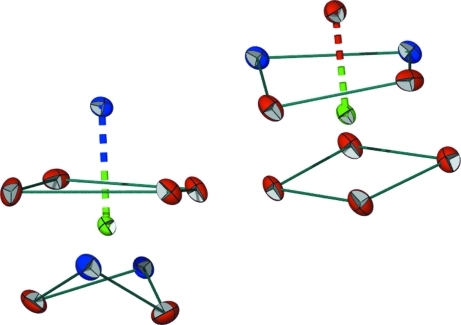
Monocapped square-antiprismatic geometry of the PrIII atoms.

## supplementary crystallographic information

### Comment

The chelating ability of the 3-aminopyrazine-2-carboxylate anion is probably similar to that of the pyrazine-2-carboxylate anion, and the crystal structures of a number of lanthanum carboxylates have been reported. The additional amino substitution in the 3-aminopyrazine-2-carboxylate should be expected to consolidate the crystal structure of the praeseodymium derivative through extensive hydrogen bonding. The synthesis of the praseodymium analog under hydrothermal conditions yielded instead the polymeric chain compound, Pr_2_(H_2_O)_5_(CHO_2_)(C_5_H_4_N_3_O_2_)_5_^.^6H_2_O; a formate group is (Scheme I, Fig. 1). In a previous synthesis, the carboxylic acid was found to decompose to an oxalate (Gao & Ng, 2011).

Adjacent chains interact with the lattice water molecules to generate a three-dimensional hydrogen-bonded network (Table 1).

### Experimental

Praeseodymium(III) nitrate hexahydrate (0.5 mmol), 3-aminopyrazine-2-carboxylic acid (2 mmol) and sodium hydroxide (2 mmol) were dissolved in water (12 ml). The solution was placed in a 23-ml, Teflon-lined Parr bomb. The bomb was heated at 433 K for 3 days. It was cooled to room temperature; colorless prismatic crystals were isolated by hand.

### Refinement

Carbon- and nitrogen-bound H-atoms were placed in calculated positions (C–H 0.93 Å, N–H 0.88 Å were included in the refinement in the riding model approximation, with *U*(H) set to 1.2*U*_eq_(C,*N*). The water H-atoms were located in a difference Fourier map, and were refined with distance restraints of O—H 0.84–0.01 Å and H···H 1.37±0.01 Å; their temperature factors were tied by a factor of 1.5 times.

The formate ion is disordered with respect to the C and uncoordinated O atoms in a 1:1 ratio; the occupancy was assumed as it could not be refined.

The final difference Fourier map had a peak/hole in the vicinity of Pr1.

Omitted from the refinement were (-1 1 12), (-1 0 3), (3 4 10), (-7 13 2), (-2 4 11), (3 3 9), (1 3 10), (-4 - 3 6), (2 4 11), (12 6 9), (-1 3 9), (1 10 13), (6 8 4), (5 7 5) and (-4 5 00.

### Crystal data

**Table:**

[Pr_2_(C_5_H_4_N_3_O_2_)_5_(CHO_2_)(H_2_O)_5_]·6H_2_O	*Z* = 2
*M**_r_* = 1215.58	*F*(000) = 1212
Triclinic, *P*1	*D*_x_ = 1.842 Mg m^−^^3^
Hall symbol: -P 1	Mo *K*α radiation, λ = 0.71073 Å
*a* = 9.7213 (3) Å	Cell parameters from 16800 reflections
*b* = 14.2113 (6) Å	θ = 3.0–27.5°
*c* = 17.6228 (6) Å	µ = 2.30 mm^−^^1^
α = 68.801 (1)°	*T* = 293 K
β = 76.291 (1)°	Prism, colorless
γ = 79.349 (1)°	0.14 × 0.12 × 0.07 mm
*V* = 2191.97 (14) Å^3^	

### Data collection

**Table:**

Rigaku R-AXIS RAPID IP diffractometer	9906 independent reflections
Radiation source: fine-focus sealed tube	8214 reflections with *I* > 2σ(*I*)
graphite	*R*_int_ = 0.041
ω scans	θ_max_ = 27.5°, θ_min_ = 3.0°
Absorption correction: multi-scan (*ABSCOR*; Higashi, 1995)	*h* = −11→12
*T*_min_ = 0.739, *T*_max_ = 0.856	*k* = −18→18
21572 measured reflections	*l* = −22→22

### Refinement

**Table:**

Refinement on *F*^2^	Primary atom site location: structure-invariant direct methods
Least-squares matrix: full	Secondary atom site location: difference Fourier map
*R*[*F*^2^ > 2σ(*F*^2^)] = 0.035	Hydrogen site location: inferred from neighbouring sites
*wR*(*F*^2^) = 0.088	H atoms treated by a mixture of independent and constrained refinement
*S* = 1.06	*w* = 1/[σ^2^(*F*_o_^2^) + (0.0227*P*)^2^ + 4.2692*P*] where *P* = (*F*_o_^2^ + 2*F*_c_^2^)/3
9906 reflections	(Δ/σ)_max_ = 0.001
679 parameters	Δρ_max_ = 1.34 e Å^−^^3^
69 restraints	Δρ_min_ = −1.03 e Å^−^^3^

### Fractional atomic coordinates and isotropic or equivalent isotropic displacement parameters (Å^2^)

**Table:**

	*x*	*y*	*z*	*U*_iso_*/*U*_eq_	Occ. (<1)
Pr1	0.41603 (2)	0.502827 (16)	0.710959 (12)	0.02425 (6)	
Pr2	0.83609 (2)	0.788695 (16)	0.719791 (12)	0.02408 (6)	
O1	0.5494 (3)	0.4496 (2)	0.59541 (18)	0.0380 (7)	
O2	0.6256 (4)	0.3460 (3)	0.5212 (2)	0.0495 (9)	
O3	0.3840 (3)	0.3776 (2)	0.84899 (17)	0.0343 (7)	
O4	0.4534 (4)	0.2648 (3)	0.9623 (2)	0.0555 (10)	
O5	0.6159 (3)	0.6033 (2)	0.63160 (17)	0.0330 (7)	
O6	0.6980 (3)	0.6774 (2)	0.70017 (17)	0.0345 (7)	
O7	1.0367 (3)	0.6542 (2)	0.72776 (17)	0.0362 (7)	
O8	1.1707 (3)	0.5181 (2)	0.79033 (18)	0.0379 (7)	
O9	0.5965 (3)	0.8007 (2)	0.79749 (19)	0.0378 (7)	
O10	0.4025 (4)	0.8696 (3)	0.8612 (2)	0.0519 (9)	
O11	0.7239 (4)	0.9123 (3)	0.6086 (2)	0.0474 (8)	
O12	0.8772 (8)	1.0092 (6)	0.5192 (4)	0.056 (2)	0.50
O12'	0.5902 (9)	0.9751 (6)	0.5161 (5)	0.059 (2)	0.50
O1W	0.2309 (3)	0.5361 (2)	0.61620 (19)	0.0372 (7)	
H11	0.162 (4)	0.571 (3)	0.635 (3)	0.056*	
H12	0.277 (4)	0.573 (3)	0.5723 (18)	0.056*	
O2W	0.4608 (3)	0.5895 (2)	0.8054 (2)	0.0387 (7)	
H21	0.392 (3)	0.634 (3)	0.812 (3)	0.058*	
H22	0.537 (3)	0.617 (3)	0.788 (3)	0.058*	
O3W	0.3224 (3)	0.6911 (2)	0.6547 (2)	0.0406 (7)	
H31	0.2358 (17)	0.699 (4)	0.675 (3)	0.061*	
H32	0.362 (4)	0.740 (3)	0.652 (3)	0.061*	
O4W	1.0343 (4)	0.8931 (3)	0.6399 (2)	0.0480 (9)	
H41	1.087 (5)	0.917 (4)	0.659 (3)	0.072*	
H42	1.005 (6)	0.939 (3)	0.600 (3)	0.072*	
O5W	0.9665 (4)	0.8011 (3)	0.82261 (19)	0.0446 (8)	
H51	1.050 (2)	0.780 (4)	0.830 (3)	0.067*	
H52	0.914 (4)	0.801 (5)	0.8676 (17)	0.067*	
O6W	0.2393 (4)	0.7251 (3)	0.85899 (19)	0.0440 (8)	
H61	0.211 (6)	0.683 (3)	0.9056 (15)	0.066*	
H62	0.272 (6)	0.772 (3)	0.865 (3)	0.066*	
O7W	0.4322 (4)	0.8556 (4)	0.6513 (4)	0.0823 (14)	
H71	0.504 (5)	0.836 (6)	0.673 (4)	0.124*	
H72	0.457 (7)	0.881 (7)	0.5998 (9)	0.124*	
O8W	0.1955 (4)	0.9725 (3)	0.7077 (3)	0.0617 (10)	
H81	0.266 (4)	0.928 (3)	0.707 (4)	0.093*	
H82	0.224 (6)	1.0301 (19)	0.691 (4)	0.093*	
O9W	0.0929 (6)	0.9679 (5)	0.8854 (4)	0.0950 (16)	
H91	0.018 (6)	0.944 (7)	0.916 (4)	0.142*	
H92	0.107 (9)	0.956 (7)	0.841 (3)	0.142*	
O10W	0.0689 (6)	1.1357 (4)	0.9393 (3)	0.0792 (13)	
H101	0.082 (9)	1.0757 (19)	0.939 (5)	0.119*	
H102	0.031 (8)	1.174 (4)	0.899 (3)	0.119*	
O11W	0.2027 (5)	1.1827 (4)	1.0380 (2)	0.0652 (11)	
H111	0.176 (6)	1.161 (5)	1.006 (3)	0.098*	
H112	0.279 (4)	1.208 (5)	1.015 (3)	0.098*	
N1	0.3688 (4)	0.3253 (3)	0.7031 (2)	0.0330 (8)	
N2	0.3064 (5)	0.1593 (4)	0.6744 (3)	0.0570 (12)	
N3	0.4764 (6)	0.1887 (4)	0.5559 (3)	0.0726 (16)	
H3A	0.4545	0.1353	0.5490	0.087*	
H3B	0.5434	0.2240	0.5197	0.087*	
N4	0.6502 (4)	0.3927 (3)	0.7672 (2)	0.0341 (8)	
N5	0.8834 (4)	0.2900 (3)	0.8402 (3)	0.0437 (10)	
N6	0.7344 (5)	0.2209 (4)	0.9634 (3)	0.0573 (12)	
H6A	0.8095	0.1892	0.9855	0.069*	
H6B	0.6484	0.2134	0.9935	0.069*	
N7	0.9158 (4)	0.7375 (3)	0.5762 (2)	0.0309 (7)	
N8	0.9749 (4)	0.6727 (3)	0.4391 (2)	0.0447 (10)	
N9	0.7825 (4)	0.5828 (3)	0.4898 (2)	0.0444 (10)	
H9A	0.8063	0.5635	0.4459	0.053*	
H9B	0.7070	0.5618	0.5274	0.053*	
N10	0.8203 (3)	0.6149 (3)	0.85557 (19)	0.0276 (7)	
N11	0.8290 (4)	0.4378 (3)	0.9913 (2)	0.0364 (8)	
N12	1.0622 (4)	0.3924 (3)	0.9406 (2)	0.0389 (9)	
H12A	1.0612	0.3381	0.9852	0.047*	
H12B	1.1400	0.4031	0.9025	0.047*	
N13	0.7367 (4)	0.9580 (3)	0.7629 (2)	0.0417 (9)	
N14	0.6270 (7)	1.1151 (4)	0.8261 (4)	0.0830 (18)	
N15	0.4277 (6)	1.0373 (4)	0.8961 (4)	0.0872 (19)	
H15A	0.3987	1.0871	0.9167	0.105*	
H15B	0.3746	0.9877	0.9099	0.105*	
C1	0.2705 (5)	0.2673 (4)	0.7555 (3)	0.0450 (11)	
H1	0.2220	0.2818	0.8029	0.054*	
C2	0.2400 (6)	0.1856 (4)	0.7402 (3)	0.0564 (14)	
H2	0.1696	0.1472	0.7775	0.068*	
C3	0.4077 (6)	0.2163 (4)	0.6214 (3)	0.0457 (12)	
C4	0.4399 (4)	0.3005 (3)	0.6368 (3)	0.0335 (9)	
C5	0.5461 (4)	0.3707 (3)	0.5795 (3)	0.0330 (9)	
C6	0.7837 (5)	0.3997 (4)	0.7244 (3)	0.0414 (11)	
H6	0.7993	0.4398	0.6688	0.050*	
C7	0.8968 (5)	0.3491 (4)	0.7610 (3)	0.0472 (12)	
H7	0.9879	0.3561	0.7292	0.057*	
C8	0.7509 (5)	0.2801 (4)	0.8845 (3)	0.0374 (10)	
C9	0.6311 (4)	0.3337 (3)	0.8464 (2)	0.0313 (9)	
C10	0.4781 (5)	0.3237 (3)	0.8901 (3)	0.0337 (9)	
C11	1.0286 (5)	0.7640 (4)	0.5170 (3)	0.0418 (11)	
H11A	1.0895	0.8048	0.5207	0.050*	
C12	1.0555 (5)	0.7305 (4)	0.4495 (3)	0.0475 (12)	
H12C	1.1354	0.7502	0.4090	0.057*	
C13	0.8612 (4)	0.6441 (4)	0.4995 (3)	0.0341 (9)	
C14	0.8306 (4)	0.6788 (3)	0.5689 (2)	0.0278 (8)	
C15	0.7060 (4)	0.6513 (3)	0.6374 (2)	0.0270 (8)	
C16	0.7085 (4)	0.5945 (4)	0.9177 (3)	0.0363 (10)	
H16	0.6265	0.6404	0.9162	0.044*	
C17	0.7145 (5)	0.5055 (4)	0.9841 (3)	0.0394 (10)	
H17	0.6344	0.4923	1.0259	0.047*	
C18	0.9451 (4)	0.4587 (3)	0.9306 (2)	0.0290 (8)	
C19	0.9370 (4)	0.5484 (3)	0.8608 (2)	0.0257 (8)	
C20	1.0576 (4)	0.5752 (3)	0.7884 (2)	0.0283 (8)	
C21	0.8085 (7)	1.0364 (5)	0.7454 (4)	0.0672 (17)	
H21A	0.8979	1.0391	0.7113	0.081*	
C22	0.7522 (9)	1.1138 (5)	0.7769 (5)	0.087 (2)	
H22A	0.8052	1.1678	0.7629	0.105*	
C23	0.5516 (7)	1.0371 (4)	0.8444 (4)	0.0566 (14)	
C24	0.6070 (5)	0.9575 (3)	0.8110 (3)	0.0353 (9)	
C25	0.5288 (4)	0.8702 (3)	0.8248 (2)	0.0331 (9)	
C26	0.757 (2)	0.9797 (17)	0.5430 (14)	0.052 (4)	0.50
H26	0.6889	1.0100	0.5095	0.063*	0.50
C26'	0.705 (2)	0.9658 (19)	0.5407 (16)	0.056 (5)	0.50
H26'	0.7791	1.0019	0.5045	0.067*	0.50

### Atomic displacement parameters (Å^2^)

**Table:**

	*U*^11^	*U*^22^	*U*^33^	*U*^12^	*U*^13^	*U*^23^
Pr1	0.02117 (11)	0.02774 (12)	0.02498 (11)	−0.00649 (8)	0.00251 (8)	−0.01252 (9)
Pr2	0.02275 (11)	0.02509 (12)	0.02534 (11)	−0.00591 (8)	−0.00256 (9)	−0.00912 (9)
O1	0.0382 (16)	0.0429 (19)	0.0378 (16)	−0.0160 (14)	0.0080 (14)	−0.0232 (14)
O2	0.054 (2)	0.050 (2)	0.0449 (18)	−0.0100 (17)	0.0141 (16)	−0.0288 (16)
O3	0.0248 (14)	0.0406 (18)	0.0325 (15)	−0.0043 (13)	−0.0009 (13)	−0.0087 (13)
O4	0.0380 (18)	0.073 (3)	0.0322 (17)	0.0019 (17)	0.0029 (15)	0.0001 (17)
O5	0.0327 (15)	0.0402 (18)	0.0332 (15)	−0.0181 (13)	0.0041 (13)	−0.0201 (13)
O6	0.0353 (16)	0.0464 (19)	0.0287 (14)	−0.0200 (14)	0.0080 (13)	−0.0220 (13)
O7	0.0266 (14)	0.0380 (18)	0.0297 (14)	0.0021 (13)	0.0053 (12)	−0.0038 (13)
O8	0.0238 (14)	0.0392 (18)	0.0336 (15)	0.0044 (13)	0.0094 (13)	−0.0052 (13)
O9	0.0328 (16)	0.0338 (17)	0.0467 (17)	−0.0052 (13)	0.0029 (14)	−0.0191 (14)
O10	0.0379 (18)	0.054 (2)	0.058 (2)	−0.0023 (16)	0.0099 (17)	−0.0246 (18)
O11	0.055 (2)	0.044 (2)	0.0390 (18)	−0.0019 (16)	−0.0176 (17)	−0.0048 (15)
O12	0.065 (5)	0.062 (5)	0.037 (4)	−0.035 (4)	−0.009 (4)	0.002 (3)
O12'	0.063 (5)	0.056 (5)	0.061 (5)	−0.006 (4)	−0.030 (4)	−0.012 (4)
O1W	0.0369 (17)	0.0442 (19)	0.0350 (16)	−0.0078 (14)	−0.0023 (14)	−0.0196 (14)
O2W	0.0364 (16)	0.044 (2)	0.0383 (16)	−0.0151 (14)	0.0091 (15)	−0.0215 (15)
O3W	0.0319 (16)	0.0350 (18)	0.0537 (19)	−0.0069 (14)	0.0018 (15)	−0.0179 (15)
O4W	0.060 (2)	0.053 (2)	0.0325 (16)	−0.0346 (19)	−0.0015 (16)	−0.0069 (15)
O5W	0.0425 (18)	0.063 (2)	0.0362 (16)	−0.0103 (17)	−0.0137 (15)	−0.0200 (17)
O6W	0.0495 (19)	0.045 (2)	0.0339 (16)	−0.0131 (16)	−0.0001 (15)	−0.0102 (14)
O7W	0.046 (2)	0.075 (3)	0.148 (4)	−0.012 (2)	−0.013 (3)	−0.063 (3)
O8W	0.052 (2)	0.050 (2)	0.091 (3)	−0.0163 (18)	−0.009 (2)	−0.030 (2)
O9W	0.100 (4)	0.092 (4)	0.097 (4)	−0.031 (3)	0.006 (3)	−0.042 (3)
O10W	0.079 (3)	0.092 (4)	0.077 (3)	0.000 (3)	−0.028 (3)	−0.036 (3)
O11W	0.067 (3)	0.085 (3)	0.0409 (19)	−0.018 (2)	−0.0025 (19)	−0.018 (2)
N1	0.0340 (18)	0.0287 (19)	0.0367 (18)	−0.0070 (15)	−0.0010 (16)	−0.0131 (15)
N2	0.068 (3)	0.044 (3)	0.064 (3)	−0.020 (2)	0.005 (2)	−0.028 (2)
N3	0.092 (4)	0.061 (3)	0.078 (3)	−0.029 (3)	0.019 (3)	−0.051 (3)
N4	0.0280 (17)	0.035 (2)	0.0351 (18)	−0.0045 (15)	0.0030 (15)	−0.0122 (16)
N5	0.0284 (19)	0.058 (3)	0.048 (2)	0.0006 (18)	−0.0078 (18)	−0.023 (2)
N6	0.044 (2)	0.078 (4)	0.039 (2)	0.006 (2)	−0.011 (2)	−0.011 (2)
N7	0.0278 (17)	0.035 (2)	0.0302 (17)	−0.0095 (15)	−0.0007 (15)	−0.0108 (15)
N8	0.039 (2)	0.065 (3)	0.0334 (19)	−0.017 (2)	0.0096 (17)	−0.0248 (19)
N9	0.046 (2)	0.064 (3)	0.0352 (19)	−0.023 (2)	0.0076 (18)	−0.032 (2)
N10	0.0255 (16)	0.0295 (19)	0.0240 (15)	−0.0035 (14)	0.0032 (14)	−0.0090 (14)
N11	0.0316 (19)	0.039 (2)	0.0300 (18)	−0.0096 (16)	0.0036 (15)	−0.0047 (16)
N12	0.035 (2)	0.036 (2)	0.0333 (18)	−0.0001 (16)	−0.0006 (17)	−0.0027 (16)
N13	0.048 (2)	0.034 (2)	0.048 (2)	−0.0094 (18)	−0.0072 (19)	−0.0193 (18)
N14	0.110 (5)	0.053 (3)	0.096 (4)	−0.016 (3)	0.009 (4)	−0.050 (3)
N15	0.092 (4)	0.066 (4)	0.100 (4)	−0.002 (3)	0.029 (4)	−0.056 (3)
C1	0.045 (3)	0.044 (3)	0.045 (3)	−0.017 (2)	0.008 (2)	−0.017 (2)
C2	0.063 (3)	0.049 (3)	0.057 (3)	−0.031 (3)	0.005 (3)	−0.016 (3)
C3	0.053 (3)	0.033 (3)	0.057 (3)	−0.005 (2)	−0.005 (2)	−0.025 (2)
C4	0.033 (2)	0.032 (2)	0.037 (2)	−0.0006 (18)	−0.0041 (19)	−0.0166 (18)
C5	0.031 (2)	0.037 (2)	0.034 (2)	−0.0028 (18)	−0.0015 (18)	−0.0181 (18)
C6	0.029 (2)	0.044 (3)	0.040 (2)	−0.002 (2)	0.008 (2)	−0.011 (2)
C7	0.027 (2)	0.056 (3)	0.054 (3)	−0.008 (2)	0.006 (2)	−0.019 (2)
C8	0.035 (2)	0.044 (3)	0.037 (2)	0.001 (2)	−0.008 (2)	−0.020 (2)
C9	0.027 (2)	0.040 (2)	0.030 (2)	−0.0026 (18)	−0.0006 (17)	−0.0192 (18)
C10	0.032 (2)	0.038 (3)	0.031 (2)	−0.0029 (19)	0.0026 (18)	−0.0164 (18)
C11	0.032 (2)	0.053 (3)	0.040 (2)	−0.018 (2)	0.003 (2)	−0.015 (2)
C12	0.039 (3)	0.064 (4)	0.033 (2)	−0.020 (2)	0.014 (2)	−0.014 (2)
C13	0.031 (2)	0.045 (3)	0.029 (2)	−0.0048 (19)	−0.0029 (18)	−0.0161 (19)
C14	0.0260 (19)	0.033 (2)	0.0241 (18)	−0.0094 (16)	0.0033 (16)	−0.0110 (16)
C15	0.0259 (19)	0.028 (2)	0.0272 (18)	−0.0037 (16)	−0.0007 (16)	−0.0117 (16)
C16	0.0233 (19)	0.044 (3)	0.033 (2)	−0.0043 (18)	0.0073 (18)	−0.0101 (19)
C17	0.032 (2)	0.047 (3)	0.029 (2)	−0.007 (2)	0.0076 (19)	−0.0084 (19)
C18	0.032 (2)	0.029 (2)	0.0247 (18)	−0.0069 (17)	0.0006 (17)	−0.0093 (16)
C19	0.0243 (18)	0.027 (2)	0.0237 (18)	−0.0042 (15)	0.0003 (16)	−0.0087 (15)
C20	0.0265 (19)	0.032 (2)	0.0232 (18)	−0.0046 (17)	0.0041 (16)	−0.0102 (16)
C21	0.067 (4)	0.054 (4)	0.088 (4)	−0.025 (3)	0.013 (3)	−0.041 (3)
C22	0.112 (6)	0.054 (4)	0.110 (6)	−0.040 (4)	0.016 (5)	−0.052 (4)
C23	0.072 (4)	0.042 (3)	0.058 (3)	0.002 (3)	−0.005 (3)	−0.028 (3)
C24	0.041 (2)	0.033 (2)	0.032 (2)	0.0028 (19)	−0.005 (2)	−0.0150 (18)
C25	0.033 (2)	0.036 (2)	0.029 (2)	0.0024 (18)	−0.0067 (18)	−0.0108 (18)
C26	0.066 (13)	0.042 (8)	0.035 (6)	−0.014 (8)	−0.010 (10)	0.006 (6)
C26'	0.054 (11)	0.055 (10)	0.042 (8)	−0.015 (8)	0.012 (9)	−0.006 (6)

### Geometric parameters (Å, °)

**Table:**

Pr1—O3	2.428 (3)	N3—C3	1.342 (6)
Pr1—O1	2.429 (3)	N3—H3A	0.8800
Pr1—O5	2.462 (3)	N3—H3B	0.8800
Pr1—O8^i^	2.478 (3)	N4—C9	1.331 (5)
Pr1—O2W	2.549 (3)	N4—C6	1.338 (5)
Pr1—O3W	2.564 (3)	N5—C7	1.333 (6)
Pr1—O1W	2.605 (3)	N5—C8	1.341 (6)
Pr1—N4	2.692 (4)	N6—C8	1.328 (6)
Pr1—N1	2.703 (4)	N6—H6A	0.8800
Pr2—O6	2.413 (3)	N6—H6B	0.8800
Pr2—O9	2.417 (3)	N7—C11	1.326 (5)
Pr2—O11	2.435 (3)	N7—C14	1.335 (5)
Pr2—O7	2.456 (3)	N8—C12	1.318 (6)
Pr2—O4W	2.482 (3)	N8—C13	1.348 (5)
Pr2—O5W	2.515 (3)	N9—C13	1.337 (5)
Pr2—N13	2.723 (4)	N9—H9A	0.8800
Pr2—N10	2.746 (3)	N9—H9B	0.8800
Pr2—N7	2.780 (3)	N10—C19	1.334 (5)
O1—C5	1.256 (5)	N10—C16	1.337 (5)
O2—C5	1.250 (5)	N11—C17	1.328 (6)
O3—C10	1.266 (5)	N11—C18	1.352 (5)
O4—C10	1.240 (5)	N12—C18	1.339 (5)
O5—C15	1.248 (5)	N12—H12A	0.8800
O6—C15	1.270 (5)	N12—H12B	0.8800
O7—C20	1.263 (5)	N13—C21	1.327 (6)
O8—C20	1.239 (5)	N13—C24	1.342 (6)
O8—Pr1^ii^	2.478 (3)	N14—C22	1.318 (9)
O9—C25	1.263 (5)	N14—C23	1.342 (8)
O10—C25	1.244 (5)	N15—C23	1.327 (7)
O11—C26'	1.20 (3)	N15—H15A	0.8800
O11—C26	1.22 (2)	N15—H15B	0.8800
O12—C26	1.243 (17)	C1—C2	1.378 (7)
O12'—C26'	1.262 (19)	C1—H1	0.9300
O1W—H11	0.84 (1)	C2—H2	0.9300
O1W—H12	0.84 (1)	C3—C4	1.420 (6)
O2W—H21	0.84 (1)	C4—C5	1.497 (6)
O2W—H22	0.84 (1)	C6—C7	1.361 (7)
O3W—H31	0.84 (1)	C6—H6	0.9300
O3W—H32	0.84 (1)	C7—H7	0.9300
O4W—H41	0.84 (1)	C8—C9	1.436 (6)
O4W—H42	0.84 (1)	C9—C10	1.510 (6)
O5W—H51	0.84 (1)	C11—C12	1.387 (7)
O5W—H52	0.84 (1)	C11—H11A	0.9300
O6W—H61	0.84 (1)	C12—H12C	0.9300
O6W—H62	0.84 (1)	C13—C14	1.427 (5)
O7W—H71	0.84 (1)	C14—C15	1.489 (5)
O7W—H72	0.84 (1)	C16—C17	1.382 (6)
O8W—H81	0.84 (1)	C16—H16	0.9300
O8W—H82	0.84 (1)	C17—H17	0.9300
O9W—H91	0.84 (1)	C18—C19	1.421 (5)
O9W—H92	0.84 (1)	C19—C20	1.500 (5)
O10W—H101	0.84 (1)	C21—C22	1.376 (8)
O10W—H102	0.84 (1)	C21—H21A	0.9300
O11W—H111	0.84 (1)	C22—H22A	0.9300
O11W—H112	0.84 (1)	C23—C24	1.422 (7)
N1—C1	1.330 (6)	C24—C25	1.489 (6)
N1—C4	1.339 (5)	C26—H26	0.9300
N2—C2	1.330 (7)	C26'—H26'	0.9300
N2—C3	1.345 (7)		
			
O3—Pr1—O1	118.90 (11)	C9—N4—C6	118.2 (4)
O3—Pr1—O5	130.60 (9)	C9—N4—Pr1	116.4 (3)
O1—Pr1—O5	67.17 (9)	C6—N4—Pr1	125.2 (3)
O3—Pr1—O8^i^	67.07 (9)	C7—N5—C8	117.3 (4)
O1—Pr1—O8^i^	141.05 (10)	C8—N6—H6A	120.0
O5—Pr1—O8^i^	141.66 (10)	C8—N6—H6B	120.0
O3—Pr1—O2W	74.58 (10)	H6A—N6—H6B	120.0
O1—Pr1—O2W	137.75 (10)	C11—N7—C14	118.6 (4)
O5—Pr1—O2W	74.37 (9)	C11—N7—Pr2	126.7 (3)
O8^i^—Pr1—O2W	81.00 (10)	C14—N7—Pr2	114.7 (2)
O3—Pr1—O3W	132.45 (10)	C12—N8—C13	116.5 (4)
O1—Pr1—O3W	108.56 (11)	C13—N9—H9A	120.0
O5—Pr1—O3W	70.21 (10)	C13—N9—H9B	120.0
O8^i^—Pr1—O3W	74.64 (10)	H9A—N9—H9B	120.0
O2W—Pr1—O3W	72.50 (11)	C19—N10—C16	118.2 (4)
O3—Pr1—O1W	120.40 (10)	C19—N10—Pr2	116.4 (2)
O1—Pr1—O1W	75.93 (10)	C16—N10—Pr2	125.3 (3)
O5—Pr1—O1W	108.77 (10)	C17—N11—C18	117.6 (4)
O8^i^—Pr1—O1W	69.75 (10)	C18—N12—H12A	120.0
O2W—Pr1—O1W	134.93 (10)	C18—N12—H12B	120.0
O3W—Pr1—O1W	67.22 (10)	H12A—N12—H12B	120.0
O3—Pr1—N4	62.15 (10)	C21—N13—C24	118.0 (5)
O1—Pr1—N4	76.64 (11)	C21—N13—Pr2	125.9 (4)
O5—Pr1—N4	73.83 (10)	C24—N13—Pr2	116.0 (3)
O8^i^—Pr1—N4	128.16 (10)	C22—N14—C23	117.2 (5)
O2W—Pr1—N4	76.48 (11)	C23—N15—H15A	120.0
O3W—Pr1—N4	137.40 (11)	C23—N15—H15B	120.0
O1W—Pr1—N4	148.53 (10)	H15A—N15—H15B	120.0
O3—Pr1—N1	70.33 (11)	N1—C1—C2	120.5 (5)
O1—Pr1—N1	61.54 (10)	N1—C1—H1	119.7
O5—Pr1—N1	127.33 (10)	C2—C1—H1	119.7
O8^i^—Pr1—N1	89.24 (11)	N2—C2—C1	123.1 (5)
O2W—Pr1—N1	144.65 (11)	N2—C2—H2	118.5
O3W—Pr1—N1	137.23 (11)	C1—C2—H2	118.5
O1W—Pr1—N1	70.05 (10)	N3—C3—N2	117.2 (5)
N4—Pr1—N1	83.46 (11)	N3—C3—C4	122.7 (5)
O6—Pr2—O9	70.23 (10)	N2—C3—C4	120.0 (4)
O6—Pr2—O11	81.60 (11)	N1—C4—C3	120.8 (4)
O9—Pr2—O11	81.77 (11)	N1—C4—C5	115.3 (4)
O6—Pr2—O7	87.91 (11)	C3—C4—C5	123.8 (4)
O9—Pr2—O7	134.68 (10)	O2—C5—O1	125.3 (4)
O11—Pr2—O7	135.14 (11)	O2—C5—C4	118.1 (4)
O6—Pr2—O4W	138.28 (10)	O1—C5—C4	116.6 (4)
O9—Pr2—O4W	141.47 (12)	N4—C6—C7	121.0 (4)
O11—Pr2—O4W	79.38 (12)	N4—C6—H6	119.5
O7—Pr2—O4W	80.18 (12)	C7—C6—H6	119.5
O6—Pr2—O5W	141.67 (11)	N5—C7—C6	123.2 (4)
O9—Pr2—O5W	98.21 (11)	N5—C7—H7	118.4
O11—Pr2—O5W	134.30 (12)	C6—C7—H7	118.4
O7—Pr2—O5W	74.59 (12)	N6—C8—N5	118.6 (4)
O4W—Pr2—O5W	72.72 (11)	N6—C8—C9	121.6 (4)
O6—Pr2—N13	127.32 (11)	N5—C8—C9	119.8 (4)
O9—Pr2—N13	61.50 (11)	N4—C9—C8	120.6 (4)
O11—Pr2—N13	72.21 (12)	N4—C9—C10	115.8 (4)
O7—Pr2—N13	141.75 (11)	C8—C9—C10	123.5 (4)
O4W—Pr2—N13	80.77 (13)	O4—C10—O3	124.9 (4)
O5W—Pr2—N13	68.18 (12)	O4—C10—C9	118.8 (4)
O6—Pr2—N10	71.21 (10)	O3—C10—C9	116.2 (4)
O9—Pr2—N10	74.03 (10)	N7—C11—C12	119.8 (4)
O11—Pr2—N10	148.30 (11)	N7—C11—H11A	120.1
O7—Pr2—N10	61.27 (9)	C12—C11—H11A	120.1
O4W—Pr2—N10	132.06 (11)	N8—C12—C11	124.2 (4)
O5W—Pr2—N10	70.47 (11)	N8—C12—H12C	117.9
N13—Pr2—N10	111.80 (11)	C11—C12—H12C	117.9
O6—Pr2—N7	61.32 (9)	N9—C13—N8	116.2 (4)
O9—Pr2—N7	125.75 (10)	N9—C13—C14	123.7 (4)
O11—Pr2—N7	68.92 (11)	N8—C13—C14	120.2 (4)
O7—Pr2—N7	67.81 (10)	N7—C14—C13	120.8 (4)
O4W—Pr2—N7	77.21 (10)	N7—C14—C15	116.1 (3)
O5W—Pr2—N7	134.98 (11)	C13—C14—C15	123.1 (4)
N13—Pr2—N7	138.04 (11)	O5—C15—O6	123.0 (4)
N10—Pr2—N7	109.50 (10)	O5—C15—C14	119.5 (3)
C5—O1—Pr1	129.5 (3)	O6—C15—C14	117.5 (3)
C10—O3—Pr1	128.6 (3)	N10—C16—C17	120.1 (4)
C15—O5—Pr1	143.6 (3)	N10—C16—H16	120.0
C15—O6—Pr2	129.7 (2)	C17—C16—H16	120.0
C20—O7—Pr2	128.3 (2)	N11—C17—C16	123.2 (4)
C20—O8—Pr1^ii^	141.7 (3)	N11—C17—H17	118.4
C25—O9—Pr2	129.8 (3)	C16—C17—H17	118.4
C26'—O11—Pr2	161.0 (11)	N12—C18—N11	117.1 (4)
C26—O11—Pr2	139.1 (9)	N12—C18—C19	123.9 (4)
Pr1—O1W—H11	104 (4)	N11—C18—C19	119.1 (4)
Pr1—O1W—H12	98 (4)	N10—C19—C18	121.7 (3)
H11—O1W—H12	110 (4)	N10—C19—C20	115.4 (3)
Pr1—O2W—H21	110 (4)	C18—C19—C20	122.9 (4)
Pr1—O2W—H22	114 (4)	O8—C20—O7	123.6 (4)
H21—O2W—H22	109 (4)	O8—C20—C19	118.7 (4)
Pr1—O3W—H31	109 (3)	O7—C20—C19	117.7 (3)
Pr1—O3W—H32	126 (4)	N13—C21—C22	120.8 (6)
H31—O3W—H32	109 (4)	N13—C21—H21A	119.6
Pr2—O4W—H41	127 (4)	C22—C21—H21A	119.6
Pr2—O4W—H42	108 (4)	N14—C22—C21	123.3 (6)
H41—O4W—H42	109 (4)	N14—C22—H22A	118.4
Pr2—O5W—H51	131 (4)	C21—C22—H22A	118.4
Pr2—O5W—H52	113 (3)	N15—C23—N14	116.8 (5)
H51—O5W—H52	110 (4)	N15—C23—C24	122.9 (5)
H61—O6W—H62	110 (4)	N14—C23—C24	120.2 (5)
H71—O7W—H72	110 (4)	N13—C24—C23	120.5 (5)
H81—O8W—H82	110 (4)	N13—C24—C25	115.4 (4)
H91—O9W—H92	109 (4)	C23—C24—C25	124.1 (4)
H101—O10W—H102	110 (4)	O10—C25—O9	123.7 (4)
H111—O11W—H112	110 (4)	O10—C25—C24	119.4 (4)
C1—N1—C4	118.3 (4)	O9—C25—C24	116.9 (4)
C1—N1—Pr1	124.5 (3)	O11—C26—O12	122.6 (18)
C4—N1—Pr1	116.8 (3)	O11—C26—H26	118.7
C2—N2—C3	117.2 (4)	O12—C26—H26	118.7
C3—N3—H3A	120.0	O11—C26'—O12'	122.8 (16)
C3—N3—H3B	120.0	O11—C26'—H26'	118.6
H3A—N3—H3B	120.0	O12'—C26'—H26'	118.6

Symmetry codes: (i) *x*−1, *y*, *z*; (ii) *x*+1, *y*, *z*.

### Hydrogen-bond geometry (Å, °)

**Table:**

*D*—H···*A*	*D*—H	H···*A*	*D*···*A*	*D*—H···*A*
O1w—H11···O7^i^	0.84 (1)	2.34 (2)	3.115 (4)	155 (5)
O1w—H12···O2^iii^	0.84 (1)	1.80 (1)	2.635 (5)	179 (5)
O2w—H21···O6w	0.84 (1)	2.02 (2)	2.839 (5)	164 (5)
O2w—H22···O6	0.84 (1)	1.99 (3)	2.771 (4)	153 (5)
O3w—H31···O7^i^	0.84 (1)	2.05 (2)	2.829 (4)	155 (4)
O3w—H32···O7w	0.84 (1)	1.89 (1)	2.721 (5)	174 (4)
O4w—H41···O8w^ii^	0.84 (1)	1.93 (1)	2.769 (5)	176 (6)
O4w—H42···O12^iv^	0.84 (1)	2.07 (5)	2.649 (7)	126 (5)
O5w—H51···O6w^ii^	0.84 (1)	1.97 (1)	2.803 (5)	174 (5)
O5w—H52···O11w^v^	0.84 (1)	1.84 (1)	2.673 (5)	173 (6)
O6w—H61···N11^vi^	0.84 (1)	2.02 (2)	2.842 (5)	169 (6)
O6w—H62···O10	0.84 (1)	2.02 (2)	2.833 (5)	164 (6)
O7w—H71···O9	0.84 (1)	2.41 (4)	3.135 (6)	146 (7)
O7w—H72···O12'	0.84 (1)	1.99 (5)	2.688 (10)	141 (8)
O8w—H81···O7w	0.84 (1)	2.01 (3)	2.782 (6)	154 (7)
O8w—H82···N2^vii^	0.84 (1)	2.03 (2)	2.861 (6)	168 (7)
O9w—H91···O10w^viii^	0.84 (1)	2.40 (6)	3.074 (8)	138 (7)
O10w—H102···N5^ix^	0.84 (1)	2.11 (3)	2.893 (6)	155 (7)
O11w—H112···O4^vii^	0.84 (1)	1.90 (1)	2.732 (5)	180 (7)
N6—H6b···O4	0.88	2.03	2.690 (5)	131
N9—H9a···O1w^iii^	0.88	2.20	2.974 (5)	147
N9—H9b···O1	0.88	2.23	3.037 (5)	152
N9—H9b···O5	0.88	2.08	2.718 (5)	129
N12—H12a···O11w^x^	0.88	2.37	3.099 (6)	140
N12—H12b···O8	0.88	2.06	2.695 (5)	129
N15—H15b···O10	0.88	2.10	2.733 (7)	129

Symmetry codes: (i) *x*−1, *y*, *z*; (iii) −*x*+1, −*y*+1, −*z*+1; (ii) *x*+1, *y*, *z*; (iv) −*x*+2, −*y*+2, −*z*+1; (v) −*x*+1, −*y*+2, −*z*+2; (vi) −*x*+1, −*y*+1, −*z*+2; (vii) *x*, *y*+1, *z*; (viii) −*x*, −*y*+2, −*z*+2; (ix) *x*−1, *y*+1, *z*; (x) *x*+1, *y*−1, *z*.
